# Low Bone Mineral Density in Men Living with HIV on Tenofovir Antiretroviral Therapy: A Cross-Sectional Exploratory Study from a Malaysian Tertiary Hospital

**DOI:** 10.3390/tropicalmed11020038

**Published:** 2026-01-29

**Authors:** Muhamad Riduan Daud, Petrick Periyasamy, Kok-Yong Chin, Najma Kori, Ummu Afeera Zainulabid, Sheng Qian Yew, Nur Ezzaty Mohammad Kazmin, Xiong Khee Cheong

**Affiliations:** 1 Infectious Disease Unit, Department of Medicine, Faculty of Medicine, Universiti Kebangsaan Malaysia, Cheras, Kuala Lumpur 56000, Malaysia; 2 Department of Pharmacology, Faculty of Medicine, Universiti Kebangsaan Malaysia, Cheras, Kuala Lumpur 56000, Malaysia; chinkokyong@hctm.ukm.edu.my; 3 Department of Internal Medicine, Kulliyyah of Medicine, International Islamic University Malaysia, Kuantan 25200, Malaysia; 4 Department of Public Health, Faculty of Medicine, Universiti Kebangsaan Malaysia, Cheras, Kuala Lumpur 56000, Malaysia; 5 Department of Internal Medicine, Faculty of Medicine and Health Sciences, Universiti Sains Islam Malaysia, Nilai 71800, Malaysia

**Keywords:** HIV, anti-retroviral therapy, tenofovir, bone mineral density, DXA scan

## Abstract

Background and objectives: Low bone mineral density (BMD) is a recognized complication in people living with HIV (PLHIV) that remains under-addressed, particularly in Malaysia. Known contributing factors for low BMD include advanced age, HIV infection itself, and prolonged use of anti-retroviral therapy (ART), particularly tenofovir-based regimens. There are limited data on the burden of low BMD in the HIV population in Malaysia. This study aimed to determine the prevalence of low BMD among men living with HIV on tenofovir disoproxil fumarate (TDF) and to identify the possible associated factors compared to a group of healthy individuals matched for age and ethnicity. Methods: This is single-center cross-sectional study involved 112 men (56 HIV-positive individuals and 56 uninfected individuals matched for age and ethnicity) who underwent dual-energy X-ray absorptiometry (DXA) scans of the femoral neck and lumbar spine. Sociodemographic, clinical lifestyle, and laboratory data, including FRAX scores for those aged more than 40 years old, were collected. Results: The prevalence of low BMD at the femoral neck in HIV-infected men on tenofovir disoproxil fumarate was significantly higher compared to healthy individuals (32.1% vs. 16.1%; *p* < 0.05). Low BMD prevalence at the lumbar spine was higher in the HIV group (8.9% vs. 3.6%; *p* = 0.463) but was not statistically significant. Older age and low body mass index (BMI) were found to be significantly associated with reduced BMD in HIV patients. Chronic kidney disease stage 2 and 3a was linked with low femoral neck BMD. HIV-related factors (duration of illness, duration of ART exposure, and CD4+ counts) showed no significant associations to low BMD. The 10-year risk of major osteoporotic and hip fractures estimated by the FRAX tool was low in both groups, and no participant exceeded the recommended intervention threshold. Conclusions: Men with HIV on tenofovir disoproxil fumarate have a higher prevalence of low BMD, particularly at the femoral neck. Traditional risk factors were more closely associated with low BMD compared to HIV-related factors and specific markers, supporting the need for routine bone health screening and preventive strategies in this population.

## 1. Introduction

According to the World Health Organization (WHO) HIV statistics report 2025, an estimated 40.8 million people were living with HIV (PLHIV) at the end of 2024, of which 3.5 million were in the WHO South-East Asia Region [1]. In Malaysia, an estimated 85,283 individuals, predominantly male, are living with HIV, with a male-to-female ratio of 8.8 to 1 [2]. Highly active antiretroviral therapy (HAART) has significantly improved the lives of PLHIV, leading to a decline in HIV-related illnesses and AIDS-related deaths [3]. The life expectancy for PLHIV is now nearly the same as for those without HIV. In fact, over 50% of PLHIV in Europe and the USA are now over 50 years [4].

HIV is a chronic infection that directly impacts bones, causing metabolic changes and reducing bone mineral density (BMD). The increasing life expectancy of PLHIV has led to increased concern about aging-related comorbidities, such as osteoporosis and fragility fractures; these conditions are now the most common complications for those on antiretroviral therapy (ART) [5]. Studies show that the overall incidence rates of fractures, osteopenia, and osteoporosis among PLHIV were significantly higher, at 21%, 43%, and 19%, respectively [6]. A meta-analysis reported that PLHIV had lower BMD at the hip (z-score −0.31; 95% CI: −0.46 to −0.27) and lumbar spine (z-score −0.36; 95% CI: −0.39 to −0.15) compared with controls [7].

Several unmodifiable and modifiable risk factors contribute to the development of low BMD in PLHIV on ART. Low BMD affects 30–60% of Asian PLHIV, with similar rates found in Caucasian and Black populations [6]. Advanced age, low body mass index (BMI), and tenofovir-based regimens are common risk factors across groups; smoking is an additional factor in Caucasians [8,9,10]. Interestingly, despite being a known predictor of low BMD, low vitamin D has not shown a statistically significant association in Asian PLHIV studies [8,11]. In addition to traditional risk factors for osteoporosis, both HIV infection and ART are independently associated with low BMD. HIV infection is known to have a negative impact on the function of endocrine glands that ultimately may increase the risk of early osteoporosis [12]. Tenofovir disoproxil fumarate (TDF) use is a known risk factor for bone demineralization. Evidence indicates TDF-containing regimens cause significant BMD loss at the hip, spine, and femoral neck [8,13,14,15], as well as increased fracture risk among younger men living with HIV [16]. This effect is highlighted by comparative prospective studies, which demonstrated that first-line TDF regimens result in a significantly greater decline in both spine and hip BMD than abacavir-containing regimens [17,18]. While the mechanism by which TDF causes bone loss is elusive, it was proposed that TDF may affect the bone indirectly through proximal tubule toxicity, resulting in phosphate wasting and increased bone turnover [19]. The complex interplay of HIV disease, current treatments, and metabolic factors significantly elevates the risk of low BMD and fractures in PLHIV.

Understanding the burden and associated risk factors for low BMD among PLHIV is needed to warrant a prevention strategy. The effect of low BMD translates to an increased risk of pathological fractures and decreased rate of fracture healing. The most common sites of fractures are the vertebrae, hip, and wrist. One of the methods to diagnose low BMD before the fracture occurs is by measuring BMD using dual-energy X-ray absorptiometry (DXA). Central DXA is considered the “gold standard” for non-invasive measurement of bone mass, in part because of its superior safety and reliability [20].

To our knowledge, there is a paucity of data on bone health among Malaysian PLHIV. Recognizing that males are the predominant population of HIV-infected individuals in Malaysia, this study aims to determine the prevalence of low BMD in men living with HIV receiving tenofovir disoproxil fumarate and its associated risk factors compared to the HIV non-infected population.

## 2. Materials and Methods

### 2.1. Ethical Consideration

Ethical approval for this study was obtained from the Universiti Kebangsaan Malaysia Research Ethics Committee (approval code: JEP-2024-665). Written informed consent was secured from all participants prior to their participation, in accordance with institutional guidelines.

### 2.2. Subjects

A single-center, cross-sectional study was conducted using purposive sampling to recruit participants from June 2024 to July 2025. The HIV group consisted of patients living with HIV who were receiving tenofovir disoproxil fumarate and were recruited from the Infectious Disease Clinic at Hospital Canselor Tuanku Muhriz (HCTM) in Kuala Lumpur, Malaysia. None were on tenofovir alafenamide (TAF). Eligible subjects were at least 21 years old and had been on ART for a minimum of one year. Exclusion criteria were a prior diagnosis of bone metabolic diseases (osteoporosis, osteomalacia, or rickets), rheumatoid arthritis, thyroid disorders, hypogonadism, and chronic kidney disease stages greater than 3a. Additionally, subjects using medications that affect bone metabolism—such as anti-osteoporosis drugs, glucocorticoids, anabolic steroids, anticonvulsants, or sex hormone replacement/deprivation therapies—were excluded. Healthy controls were recruited from HCTM staff during the same period and had no prior HIV diagnosis or ART exposure. The same inclusion and exclusion criteria outlined above applied to this group.

### 2.3. Sample Size

The sample size was calculated on the basis of the estimated 90% confidence interval, with population size taken from the previous study [10]. By using the formula with Kish’s formula, with prevalence of 0.192 and margin of error 10%, the sample size was 52 for each group. The study was designed as an exploratory cross-sectional analysis to provide preliminary estimates and identify potential associations rather than to generate highly precise prevalence estimates.

### 2.4. Data Collection

After obtaining written informed consent, we collected data on clinical history, comorbidities, duration of HIV infection and ART exposure, current medications, and lifestyle factors such as smoking and alcohol use through interviews and medical records. Regular alcohol consumption was defined as an intake of at least 3 units per day. Dietary calcium intake and fracture history were recorded, with a high calcium diet defined as self-reported daily consumption of two or more servings of dairy or calcium-rich foods, in line with international dietary guidelines.

For the HIV group, recent laboratory results (within the past three months) were extracted, including CD4 count, HIV RNA level, HbA1c, renal profile, and lipid profile. Laboratory tests were not required for the control group. Body weight and height were measured with a digital scale and stadiometer, and BMI was calculated as weight in kilograms divided by height in meters squared.

All participants underwent dual-energy X-ray absorptiometry (DXA) at the lumbar spine (L1–L4) and left femoral neck using a Hologic Discovery QDR Wi densitometer. Scans were performed by a trained operator blinded to group allocation, and the same protocol was used throughout the study. T- and Z-scores were calculated using sex- and ethnicity-matched reference data. For this study, low BMD was defined as osteoporosis or osteopenia (T-score ≤ −1.0) in men aged 50 years or older, or a Z-score ≤ −2.0 (below the expected range for age) in men younger than 50 years, at either the femoral neck or lumbar spine. For participants aged ≥40 years, FRAX was used to estimate 10-year fracture risk, with results expressed as the percentage probability of a fracture occurring over the next 10 years.

### 2.5. Data Analysis

Data analysis was conducted using SPSS version 30.0 (IBM Corp., Armonk, NY, USA). Categorical data are expressed as frequencies and percentages. Continuous variables with normal distribution are expressed as means ± standard deviation, whereas non-normally distributed variables are presented as medians with interquartile ranges (IQR, 25th–75th percentiles). BMD values (g/cm^2^) were analyzed as continuous variables in correlation analyses, whereas low BMD status (normal vs. low BMD) was treated as a dichotomous outcome for prevalence and categorical association analyses. Baseline characteristics and BMD values between the HIV and healthy groups were compared with independent-sample *t*-tests for parametric data, the Mann–Whitney U test for non-parametric data, and the Chi-square or Fisher’s exact test for categorical data. The prevalence of low BMD between groups was assessed using the Chi-square test.

Further analyses aimed to identify factors associated with low BMD among HIV subjects. Bivariate correlations examined relationships between continuous predictors (age, BMI, biochemical parameters, and HIV-related factors) and BMD values using Pearson’s or Spearman’s correlation, depending on data distribution. Bivariate analyses were exploratory and intended to identify associations rather than causal relationships. For categorical predictors (diabetes mellitus, hypertension, dyslipidemia, lifestyle factors, and comorbidities), associations with BMD status (low vs. normal) were evaluated with Chi-square or Fisher’s exact tests. For participants aged ≥40 years, FRAX scores were reported as percentages and compared between groups using independent *t*-tests. A two-tailed *p*-value < 0.05 was considered statistically significant.

## 3. Results

**Figure 1:**
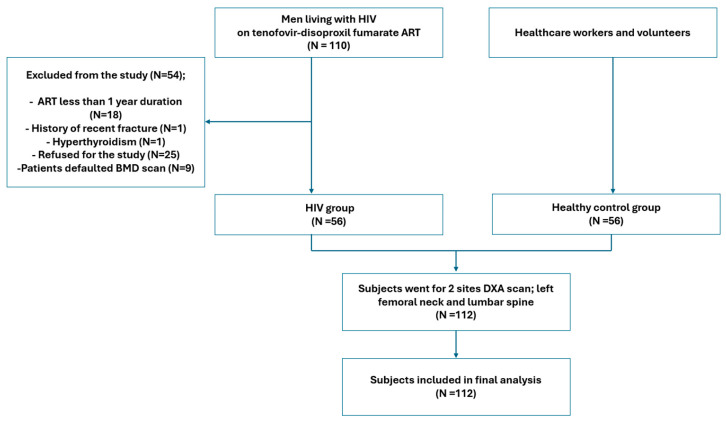
Study flow chart.

A total of 345 PLHIV who were receiving tenofovir disoproxil fumarate were under Infectious Disease Clinic HCTM follow up during the study period. Of these, 110 men patients were screened for the study. A total of 54 patients were excluded, in which 25 patients refused to join the study, 18 were receiving ART for less than one year, 1 had a fracture within a year, another patient had hyperthyroidism, and 9 patients defaulted on their BMD scan appointment. As a result, only 56 patients were included in the final analysis. For comparison, a total of 56 age- and sex-matched healthy individuals were recruited as healthy controls. The study flow chart is shown in Figure 1.

### 3.1. Sociodemographic and Clinical Characteristics

The HIV and healthy control groups were comparable in terms of baseline sociodemographic and clinical characteristics, as shown in Table 1. Both groups were comparable in age, ethnicity, body mass index (BMI), smoking status, and alcohol consumption, with no statistically significant differences observed. About one-quarter of participants in both groups were older than 50 years, with no significant difference between them. Ethnic distribution was similar between the two groups, Malays were the majority in both groups, followed by Chinese and Indian participants. Similarly, there was no significant difference in mean BMI. Lifestyle factors such as smoking and alcohol consumption were slightly higher among the HIV group, but these differences were not statistically significant. A high calcium diet habit showed a trend towards lower consumption in the HIV group. In terms of comorbidities background, the prevalence of dyslipidemia, chronic kidney disease (CKD), and fatty liver disease was significantly higher in the HIV group. Other comorbidities, like hypertension and diabetes, were less common. Among HIV individuals, the median age of diagnosis of HIV duration and exposure to ART were less than 10 years, with 85% of them having a good CD4+ cell count. All of them were virological suppressed.

### 3.2. Bone Mineral Density

There were no significant differences in the mean BMD of the femoral neck and lumbar spine between HIV and healthy controls, as shown in Table 2. However, the HIV group had a higher prevalence of low BMD at the femoral neck (32.1% vs. 16.1%; *p* = 0.047), while the prevalence based on lumbar spine did not show significant difference between the two groups (*p* = 0.463).

For the femoral neck, 18 subjects from the HIV groups and 8 subjects from the healthy group showed low BMD. For the lumbar spine, low BMD was observed among five subjects in the HIV group and only two subjects in the healthy group. Among HIV subjects aged <50 years old, 38.9% HIV subjects showed a Z-score ≤ −2.0, compared with 12.5% in healthy controls. In participants aged ≥ 50 years, osteopenia (T-score −1.0 to −2.5) was observed in 44.4% of HIV patients and 75% of controls, while osteoporosis (T-score ≤ −2.5) occurred in 16.7% and 12.5%, respectively. At the lumbar spine, 20% of HIV participants aged <50 years showed a Z-score ≤ −2.0, while none of the controls were below the expected range for age. Among those aged ≥ 50 years, osteopenia was found in 80% of HIV patients and 100% of controls, with no cases of osteoporosis detected at this site. This summary of low BMD scores is shown in Table 3.

### 3.3. Associations for Possible Risk Factors to Low BMD

Bivariate correlation analysis (Pearson’s or Spearman’s correlation) was performed to examine the association between low BMD and continuous independent variables (Table 4). The analysis found that older age was significantly associated with lower BMD at the femoral neck and lumbar spine. BMI was negatively correlated with femoral neck BMD, indicating that a lower BMI was associated with reduced bone mineral density. Other clinical and laboratory parameters, including duration of HIV illness and exposure to ART and CD4+ counts and individual laboratory parameters, were not significantly correlated with low BMD at either site of measurement. CKD stages 2 and 3a were significantly associated with low femoral neck BMD. For the lumbar spine, hypertension was the only factor significantly associated with lower BMD. No significant associations were observed with other categorical factors (Table 5).

### 3.4. FRAX Calculation Scores

The 10-year probability of fracture was calculated using FRAX scores for participants aged 40 and above. The comparison showed no statistically significant difference in fracture risk between HIV patients and healthy individuals, as shown in Table 6.

## 4. Discussion

This study observed a higher proportion of low BMD in men living with HIV compared to age- and ethnicity-matched healthy controls; however, this finding should be interpreted cautiously given the exploratory design and limited precision of the prevalence estimate. The femoral neck was the most common site where the prevalence of low BMD in the HIV group was nearly double that of the healthy controls (32.1% vs. 16.1%). Although most studies and a meta-analysis by Afraie et al. (2025) reported a higher prevalence of low BMD at the lumbar spine, our findings aligned with a study from Thailand, which showed the femoral neck as the prominent site of low BMD in men with HIV compared to HIV-negative men (62% vs. 44%) [6,8,10,21]. Studies reported that 15 to 60% had low BMD in PLHIV [8,10,21,22,23]. The majority of HIV individuals in this study had high CD4 count and were virologically suppressed. These findings are consistent with some studies which suggest that HIV infection itself was associated with low BMD [24,25]. Collectively, these findings suggest an association between HIV infection and lower BMD, even among individuals with good virological control. However, in the absence of multivariate analyses and longitudinal data, a direct or independent effect of HIV on BMD cannot be established.

This study found that older age was positively correlated with lower BMD at both the femoral neck and lumbar spine in men living with HIV. It was consistent with the a meta-analysis reported by Afraie et al. (2025), identifying advanced age of more than 50 years old as a traditional risk factor across Caucasian, Black, and Asian populations of PLHIV [6]. The association between older age and low BMD in this population is likely multifactorial and may reflect the combined effects of aging and HIV-related chronic inflammation.

The low testosterone and estrogen levels in elderly and HIV populations directly impacts the bone remodeling [26]. Chronic inflammation in HIV has been associated with dysregulation of osteoblast and osteoclast activity, which may contribute to reduced bone mineral density [27]. The inflammatory process persists despite being on ART, causing increased bone resorption. This effect may be more pronounced with advancing age, as aging itself is associated with increased inflammatory activity.

Lower BMI correlated negatively with femoral neck BMD in our study. A previous study in Kosovo demonstrated a significant positive correlation between weight and femur neck BMD (r = 0.445; *p*-value < 0.01) [28]. Higher BMI has been associated with higher BMD, potentially reflecting greater mechanical loading on bone and adaptive bone remodeling. Additionally, a higher BMI can lead to increased insulin and leptin levels, which may also contribute to higher BMD. Conversely, low BMI may reflect reduced mechanical loading, which has been associated with lower bone mineral density. This study found the association to be site-specific, only observed in the femoral neck but not in the lumbar spine. This divergence suggests that the influence of body mass on bone health in this population may differ between skeletal sites. While a simple correlation in a previous Iranian study showed an association between BMI and lumbar spine BMD, this relationship was not confirmed in a subsequent regression analysis after controlling for other variables [29]. Lumbar spine BMD can be artificially elevated due to artefacts. Elderly and overweight individuals are more susceptible in developing degenerative spine changes, such as osteophytes and facet arthritis [30]. These artefacts affect the lumbar spine BMD reading; thus, it is less reflective of the true bone status and may mask the true association between BMI and lumbar spine BMD.

Chronic kidney disease (CKD), in particular stage 3b and above (eGFR below 45 mL/min/1.73 m^2^), is a well-known risk factor for low BMD in the general population and HIV individuals due to CKD mineral bone metabolism (CKD-MBD). CKD–mineral bone disorder has been associated with abnormalities in parathyroid hormone regulation, phosphate balance, and vitamin D and calcium metabolism, which are linked to increased bone resorption. In PLHIV, these mechanisms may be further influenced by ART-related toxicity, including tenofovir-associated tubular dysfunction, which has been linked to reduced bone mineral density [31]. Interestingly, we found that early CKD, stages 3a and 2 (eGFR range 45 to 90 mL/min/1.73 m^2^), had a significant correlation with low BMD at the femoral neck. In 2016, Streinu-Cercel, Săndulescu et al. reported that about 30% of the study population had stage 2 CKD, and many of these exhibited lumbar or femoral osteopenia/osteoporosis, highlighting the overlap between early CKD and reduced BMD in the HIV cohort [32]. A study by Bezzera et al. (2019) has shown that, even in early CKD stages (eGFR 45 to 90 mL/min/1.73 m^2^, corresponding to stages 2 and 3a), there was a significant correlation with low BMD at the femoral neck in the general population [33]. Compared with the general population, where CKD–mineral bone disorder is typically more evident in advanced CKD, people living with HIV may experience earlier bone involvement, potentially reflecting the combined effects of ART exposure and HIV-related inflammation.

The duration since the HIV diagnosis, and the exposure to tenofovir-based ART and CD4 count, did not show a significant association with low BMD in this study. This finding contrasts with a meta-analysis that reported a higher risk of low BMD with an HIV duration and ART exposure of more than 10 years [6]. Bone loss has been observed to occur most rapidly during the first 6–12 months following ART initiation, with some studies reporting continued decline beyond the first year. The START sub-study demonstrated that lumbar spine BMD stabilized after the first year of antiretroviral therapy, while hip BMD continued to decline for up to three years [34]. On the other hand, some literature has reported that the CD4 count, HIV viral load, the length of time since the HIV diagnosis, and antiretroviral therapy were not predicting factors for low BMD [29]. In our HIV cohort, the median duration since the HIV diagnosis was 6 years (IQR 4, 8), and duration of ART exposure was 5 years (IQR 4, 7.8) years; this relatively shorter duration may partly account for the absence of a significant association with low BMD in our cohort, as a longer duration since the HIV diagnosis appears to confer greater risk in other studies. Furthermore, majority of HIV individuals in this study had a CD4 of more than 200 cells/μL, which may have limited the capacity to show the association between them.

The fracture risk assessment tool (FRAX) is recommended for the assessment of the 10-year fracture risk in HIV individuals older than 40 years old in some guidelines [35]. However, it may underestimate fracture risk in PLHIV because this algorithm does not fully capture HIV-specific risk factors compared to other traditional risk factors. In our study, the calculated 10-year risk of major osteoporotic and hip fractures using the FRAX tool was generally low in both men living with HIV and the healthy group, with no subjects exceeding commonly accepted intervention thresholds. These findings are comparable with a previous study by Womack et al. (2023), who demonstrated that FRAX underestimated the risk of osteoporosis in PLWHIV, as most individuals with low BMD usually present with normal FRAX scores, suggesting that FRAX alone may have limited utility in this population [36]. Another a study performed by Vizcarra et al. (2020) showed FRAX tends to underestimate fracture risk when HIV is not accounted for as a secondary cause of osteoporosis [37]. The low FRAX scores in our study may be explained by the relatively younger age group of our participants, their generally healthy body weight, and the low number of traditional fracture risk factors of hip fractures. This shows that FRAX alone may not be adequate for predicting fracture risk in people with HIV because it does not include HIV-related factors, such as duration of disease, their immune status, or duration of therapy. Therefore, even though the FRAX scores were low, bone density scans are still an important tool for bone health screening in HIV populations. There is a need to improve the existing screening fracture risk tools so that they better reflect HIV-specific risks.

This study was the first study to compare bone mineral density between men with HIV and healthy men in Malaysia. Interestingly, this study identified an association between early-stage kidney disease and low bone mineral density among men living with HIV. While other studies have shown that older age and low BMI are important risk factors for developing low BMD, our study adds useful information for the HIV population in Asia. This study has several limitations. In addition, the modest sample size and the use of a wide margin of error limit the precision of the prevalence estimates; therefore, the reported prevalence figures should be interpreted as preliminary and hypothesis-generating rather than definitive. The cross-sectional design of this study prevents the establishment of causal relationships between HIV, ART, and BMD outcomes. This study did not include ART-naïve individuals or patients receiving TAF-based regimens; therefore, the differential effects of ART initiation and tenofovir formulations on BMD could not be evaluated. As a single-center study with a small sample size, its findings have limited generalizability to the wider population. Furthermore, the sample size was too small to perform a multivariate analysis. Our study also did not account for key modifiable factors known to influence BMD. For instance, a detailed history of regular physical activity, which is important for analyzing bone health, was not collected. Similarly, vitamin D status and parathyroid hormone levels, which are critical for bone metabolism, were not included. This omission further limited a comprehensive assessment of bone health. Furthermore, the lack of laboratory data for the control group in this study could result in the underdiagnosis of comorbidities, including diabetes mellitus and kidney disease.

Future research should prioritize large, multi-center randomized studies to ensure the results are widely generalized. A longitudinal study design is crucial to elucidate the trajectory of BMD changes and to differentiate the causal contributions of the HIV virus versus ART-induced bone demineralization. Comparing tenofovir- with non-tenofovir-based ART regimens would clarify how different treatments affect bone health. Moreover, study populations must be diversified to include women and older adults—groups with an inherently higher risk for osteoporosis and fractures. To better understand the underlying pathophysiology of low BMD in HIV, future studies should incorporate the measurement of key mediators such as vitamin D, sex hormones, and bone turnover markers. Interventional trials assessing the efficacy of dietary modifications, lifestyle adjustments, or strategic ART switches are also warranted. Lastly, improving the accuracy of clinical risk stratification requires the refinement and validation of predictive tools such as FRAX for specific use in Asian populations living with HIV.

## 5. Conclusions

Men living with HIV receiving tenofovir-based antiretroviral therapy in this Malaysian tertiary care setting were observed to have a higher proportion of low bone mineral density, particularly at the femoral neck, compared with age- and ethnicity-matched healthy individuals. However, given the exploratory cross-sectional design and the limited precision of the prevalence estimates, these findings should be interpreted cautiously and regarded as preliminary. Our results suggest that reduced BMD may be present in men living with HIV on tenofovir-based ART. While these findings support further investigation into bone health in this population, larger and adequately powered studies are needed before firm conclusions can be drawn or definitive recommendations regarding routine DXA screening can be made. Traditional risk factors such as older age and lower body mass index appeared to be more consistently associated with reduced BMD than HIV-specific parameters in this cohort. Although fracture risk estimates using FRAX were low in both groups, this tool may underestimate fracture risk in people living with HIV. Therefore, FRAX should be interpreted cautiously and considered complementary rather than definitive in this population. Until more robust evidence is available, clinicians should remain attentive to bone health in men living with HIV, particularly those with established osteoporosis risk factors, while awaiting data from larger longitudinal and multi-center studies.

## Figures and Tables

**Table 1:** Sociodemographic and clinical characteristics of the study populations.

Characteristics	HIV Group (n = 56)	Healthy Group (n = 56)	*p*-Value
	Value	Value	
Age (years) Mean ± SD	43.29 *±* 8.16	43.71 *±* 9.21	0.795 *
Age ≥ 50 years old N (%)	14 (25)	16 (28.6)	0.831 **
Age < 50 years old N (%)	42 (75)	40 (71.4)	
Ethnicities N (%)			
Malay	36 (64.3)	30 (53.6)	
Chinese	17 (30.4)	22 (39.3)	0.564 **
Indian	3 (5.4)	4 (7.1)	
BMI (kg/m^2^) Mean **±** SD	25.4 *±* 4.5	25.1 *±* 3.5	0.663 *
Smoking status N (%)	15 (26.8)	10 (17.9)	0.257 **
Regular alcohol user N (%)	8 (14.3)	10 (17.9)	0.607 **
Previous fracture N (%)	2 (3.6)	5 (8.9)	0.438 ^#^
High-calcium diet N (%)	0	5 (8.9)	0.057 ^#^
Time since HIV diagnosis (years ) Median (IQR)	6 (4, 8)	-	-
Duration of tenofovir-based ART (years) Median (IQR)	5 (4, 7.8)	-	-
Nadir CD4+ counts (cells/µL) Median (IQR)	177 (24.3, 346.5)		
CD4+ counts (cells/µL) Median (IQR)	457 (297, 673)	-	-
CD4+ < 200 cells/µL N (%)	8 (14.3)	-	-
CD4+ > 200 cells/µL N (%)	48 (85.7)	-	-
HIV viral load (copies/mL)	ND	-	-
Other comorbidities N (%)			
CKD stages 2 and 3a	36 (64.3)	0	<0.001 **
Dyslipidemia	25 (44.6)	6 (10.7)	<0.001 **
Fatty liver	7 (12.5)	0	0.013 ^#^
Hypertension	4 (7.1)	7 (12.5)	0.341 **
Diabetes mellitus	2 (3.6)	0	0.495 ^#^
Heart disease	2 (3.6)	0	0.495 ^#^
Laboratory investigations			
Hemoglobin A1c (%) Median (IQR)	5.45 (5.2, 5.7)	-	-
Creatinine (umol/L) Mean ± SD	90.14 ± 16.10	-	-
EGFR (mL/min/1.73 m^2^) Mean ± SD	83.18 ± 17.77	-	-
Serum cholesterol (mmol/L) Mean ± SD	5.19 ± 0.89	-	-
LDL cholesterol (mmol/L) Mean ± SD	3.36 ± 0.84	-	-
Triglyceride (mmol/L) Median (IQR)	1.55 (1.09, 2.00)	-	-
Alkaline phosphatase (µ/L) Median (IQR)	103.5(92.3, 129.8)	-	-

* Independent *t*-test; ** Chi-square test; ^#^ Fisher’s exact test. SD: standard deviation; IQR: interquartile range; BMI: body mass index; CKD: chronic kidney disease; EGFR: estimated glomerular filtration rate; CKD 2 stage 2 is defined by EGFR 60–89 mL/min/1.73 m^2^ and CKD stage 3a is defined by EGFR 45–59 mL/min/1.73 m^2^; LDL: low-density lipoprotein; ND: viral load level not detected (<40 copies/mL); “-”: data not determined.

**Table 2:** Bone mineral density measurements and proportion of low BMD among HIV and healthy groups.

Site of Bone	Variable	HIV Group (n = 56)	Healthy Group (n = 56)	*p*-Value
Femoral neck	Mean BMD (g/cm^2^) Mean ± SD	0.75 ± 0.11	0.78 *±* 0.12	0.157 *
	Low BMD prevalence (%)	32.1	16.1	0.047 **
Lumbar spine	Mean BMD (g/cm^2^) Mean ± SD	0.97 ± 0.13	1.00 ± 0.13	0.222 *
	Low BMD prevalence (%)	8.9	3.6	0.463 **

BMD: bone mineral density; SD: standard deviation; * Fisher’s exact test; ** Chi-square test.

**Table 3:** Summary of low BMD based on age group in the study population.

Age	Low BMD	HIV Group	Healthy Group	Category of Low BMD
Femoral neck		n = 18 (100%)	n = 8 (100%)	
Age < 50 years old	Z-score ≤ −2.0	7 (38.9)	1 (12.5)	Below the expected range for age
Age ≥ 50 years old	T-score −1.0 to −2.5	8 (44.4)	6 (75)	Osteopenia
	T-score ≤ −2.5	3 (16.7)	1 (12.5)	Osteoporosis
Lumbar spine		n = 5 (100%)	n = 2 (100%)	
Age < 50 years old	Z-score ≤ −2.0	1 (20%)	0	Below the expected range for age
Age ≥ 50 years old	T-score −1.0 to −2.5	4 (80%)	2 (100%)	Osteopenia
	T-score ≤ −2.5	0	0	Osteoporosis

Low BMD: Z-score ≤ −2.0 (below expected range for age), T-score −1.0 to −2.5 (osteopenia), and T-score ≤ −2.5 (osteoporosis).

**Table 4:** Bivariate correlation analysis between low BMD at either the femoral neck or the lumbar spine among HIV individuals and potential associated risk factors.

Variables	Femoral Neck		Lumbar Spine	
	Correlation Coefficient	*p*-Value	Correlation Coefficient	*p*-Value
Age *	0.286	0.002	0.297	0.026
Body mass index (BMI) *	−0.213	0.024	−0.460	0.738
Time since HIV diagnosis ^#^	0.029	0.834	−0.033	0.808
CD4 counts ^#^	−0.024	0.863	−0.010	0.944
Nadir CD4 counts ^#^	0.064	0.640	−0.045	0.744
Duration of ART exposure ^#^	0.117	0.391	−0.059	0.668
Total serum cholesterol *	−0.018	0.894	−0.145	0.286
Serum LDL level *	−0.034	0.804	−0.136	0.318
Triglyceride level ^#^	−0.025	0.856	0.126	0.355
Hba1c level ^#^	0.157	0.248	0.064	0.638
eGFR value *	−0.152	0.263	0.014	0.919
Alkaline phosphatase ^#^	0.159	0.243	0.430	0.755

* Pearson’s correlation; ^#^ Spearman’s correlation.

**Table 5:** Association between categorical risk factors and low BMD at femoral neck and lumbar spine among HIV patients.

Variables	Femoral Neck *p*-Value	Lumbar Spine *p*-Value
Smoking	0.908 **	0.309 ^#^
Alcohol intake	0.703 ^#^	0.552 ^#^
CD4+ < 200 cells/µL	1.000 ^#^	0.552 ^#^
Diabetes mellitus	0.544 ^#^	1.000 ^#^
Hypertension	0.587 ^#^	0.036 ^#^
Dyslipidemia	0.388 **	0.647 ^#^
CKD stages 2 and 3a	0.041 ^#^	0.645 ^#^
Heart disease	1.000 ^#^	1.000 ^#^
Fatty liver	0.669 ^#^	1.000 ^#^
History of previous fracture	0.099 ^#^	0.172 ^#^

** Chi-square test; ^#^ Fisher’s exact test; CKD: chronic kidney disease.

**Table 6:** Comparison of FRAX scores between subjects with HIV and healthy controls above 40 years old.

HIV Patients (n = 35)	Normal Patients (n = 34)	*p*-Value
FRAX Score (Hip Fracture) %		
0.417 (0.623)	0.335 (0.375)	0.497 *
FRAX Score (Major Fracture) %		
1.494 (1.492)	1.432 (0.956)	0.780 *

* Independent *t*-test.

## Data Availability

Data are available from the authors at reasonable request.

## Abbreviations

The following abbreviations are used in this manuscript:

WHO	World Health Organization
HIV	Human Immunodeficiency Virus infection
PLHIV	People living with Human Immunodeficiency Virus infection
AIDS	Acquired immunodeficiency syndrome
HAART	Highly active anti-retroviral therapy
ART	Anti-retroviral therapy
TAF	Tenofovir alafenamide
TDF	Tenofovir disoproxil fumarate
BMI	Body mass index
BMD	Bone mineral density
DXA	Dual-energy X-ray absorptiometry
RNA	Ribonucleoside acid
CKD	Chronic kidney disease
LDL	Low-density lipoprotein
SD	Standard deviation
IQR	Interquartile range
